# Kinetic Modeling of a Heterogeneous Fenton Oxidative Treatment of Petroleum Refining Wastewater

**DOI:** 10.1155/2014/252491

**Published:** 2014-01-29

**Authors:** Diya'uddeen Basheer Hasan, Abdul Aziz Abdul Raman, Wan Mohd Ashri Wan Daud

**Affiliations:** ^1^Department of Chemical Engineering, Faculty of Engineering, University of Malaya, 50603 Kuala Lumpur, Malaysia; ^2^National Research Institute for Chemical Technology, PMB 1052, Zaria, Nigeria

## Abstract

The mineralisation kinetics of petroleum refinery effluent (PRE) by Fenton oxidation were evaluated. Within the ambit of the experimental data generated, first-order kinetic model (FKM), generalised lumped kinetic model (GLKM), and generalized kinetic model (GKM) were tested. The obtained apparent kinetic rate constants for the initial oxidation step (*k*_2_′), their final oxidation step (*k*_1_′), and the direct conversion to endproducts step (*k*_3_′) were 10.12, 3.78, and 0.24 min^−1^ for GKM; 0.98, 0.98, and nil min^−1^ for GLKM; and nil, nil, and >0.005 min^−1^ for FKM. The findings showed that GKM is superior in estimating the mineralization kinetics.

## 1. Introduction

Petroleum Refinery Effluent (PRE) is refractory wastewater composing of complex aromatics organic and inorganic constituents [1]. Pollutants from refineries have been identified as highly toxic and comparatively more refractory to natural degradation compared to other industrial streams [2, 3]. There are literature reports on the treatment of this category of wastewater by the traditional processes, namely, coagulation, flocculation, membrane, adsorption, and others [4–6]. However, the biological process is the most widely used on industrial scale [1]. Generally, these processes are not ideal as they only succeed in contaminants transfer from one phase to another or partially degrade PRE [2, 7]. Among the problems of these conventional methods are high treatment and disposal cost of the produced sludge and high electrical consumption due to use of UV lamps and pumps [4, 8].

A treatment method proven to be effective in treatment of varied refractory-containing organic wastewaters is Advanced Oxidation Processes (AOPs) [2, 3, 9]. Basically, the efficiency of the oxidative processes is driven by highly reactive free hydroxyl radicals (^∙^OH) which are generated in situ [10, 11]. In this regard, Fenton and Fenton-like oxidation, which fall under the category of AOPs, have been well proven to effectively mineralize and degrade a variety of refractory organics in water [10, 12–14]. Fenton oxidative processes merit among others as they do not produce any toxic substances in water environment [12]. In addition, the process is commonly used due to the simplicity of the equipment, safe operation, minimal sludge generation, high organic destruction efficiency, and readily available reagents [3, 15–17]. ^∙^OH is generated by decomposition of hydrogen peroxide with a transition metal catalyst in Fenton oxidative processes [18]. Fenton reactions are complex, but it can be represented with a cyclic loop starting with oxidation of ferrous iron(II) to ferric iron(III) and closing with reduction of iron(III) to iron(II) by the same hydrogen peroxide [19].

Generally, iron ions at acidic conditions are mostly employed. In the case of utilizing Fe^2+^ as the catalyst source, the process is referred to as classical Fenton oxidation. On the other hand, when other transition metals are used, the oxidation becomes Fenton-like. Notable transition metals used are Fe^2+^, Cu^2+^, Mn^2+^, and Ag^+^. However, classical Fenton oxidation is widely adopted as Fe^2+^ shows superior catalytic activity in comparison to the other transition metals [20]. However, a form of heterogeneous Fenton-like system that uses zerovalent nanoparticles iron (nZVI) catalysts (Fe^0^) has been found to be efficient in the remediation of polluted wastewater. This is mainly due to their large specific surface area, high surface reactivity, and ability to penetrate into zones that are inaccessible to microsize solid catalysts. This may account for the observed improved mineralisation efficiency and biodegradability of Fe^0^ over Fe^2+^ reported by Khan and co-workers [21].

Despite numerous literature related to Fenton oxidation of recalcitrant waste streams, data is scarce on its application to PRE treatment. Specifically, the literature reports are limited to those completed by Coelho et al. [22] and recently by our group [23]. However, the process kinetics were not addressed in both works. This is understandable considering that, regardless of abundant research on Fenton reaction, the reaction mechanism and kinetics of Fenton reaction have seldom been investigated in detail [24]. The study of process kinetics is very significant and a key component in the design of industrial units [13]. Therefore, a study of PRE Fenton oxidation kinetics would provide a guide on application of Fenton oxidation. Based on the literature review, there is an overall lack of information on kinetics of PRE mineralisation by Fenton oxidation or other advanced oxidation processes. Thus, establishing a kinetic model is very important.

The specific objective of this study is to investigate the Fenton oxidative mineralisation kinetics of PRE in the context of providing insight into the reaction process and experimentally establishing the most suitable model that represents the treatment process. nZVI heterogeneous catalyst (Fe^0^) was employed at the optimised parameters for the mineralisation of the PRE [23]. Kinetic studies were conducted on the basis of the mineralisation pathway suggested in the literature.

## 2. The Fenton Equations 

There are different postulations to the number of groups forming the main Fenton oxidation [25]. However, most studies have reported that the generally accepted Fenton oxidative reactions involve few main reactions which are divided into two groups. First, the reaction of inorganic species such as Fe^0^, Fe^2+^, Fe^3+^, H_2_O_2_,^∙^OH, and HO_2_^∙^ (reaction (1)) [11]:

(1) Fe0+H2O2+2H+⟶Fe2++2H2OFe2++H2O2⟶Fe3++OH−+OH∙Fe3++H2O2⟶Fe2++HO2∙+H+Fe3++HO2∙⟶  Fe2++O2+H+.

Second, the reaction of reactive species in the first group with the organic species (comprising of the contaminants and byproducts) (reaction (2)):

(2) RH+OH∙⟶R∙+H2OR∙+Fe3+⟶R∙+Fe2+R∙+Fe2+⟶R∙+Fe2+R∙+H2O2⟶ROH+OH∙R∙+O2⟶ROO∙.

Other reactions, such as side and scavenging reactions, are depicted in reaction (3) [10, 26, 27]. These reactions are known to always coexist along with the main reactions [12]. However, they are assumed to have negligible influence on the reaction system in stoichiometrically conducted experiments [23]:

(3) H2O2+OH∙⟶H2O+HO2∙HO2∙+OH∙⟶H2O+O2OH∙+OH∙⟶H2O2Fe2++OH∙⟶Fe3++OH−.

Fenton reactions have been extensively discussed in the literature [25] and the basic assumption of the models generated from these reactions can be summarised as

- solution in the batch reactor is completely mixed;
- temperature is constant;
- value of pH decreases slightly during the process. However, as the variation mainly affects the Fe species and Fe^2+^ is the dominant form of Fe(II) species at pH 2.6–3.0, these ranges are not affected by the pH change and thus these effects are neglected;
- fluctuation in concentrations of H_2_O_2_ can be safely assumed to be constant. This is against the backdrop that ^∙^OH is a highly reactive free radical with an extremely short lifetime of nanoseconds. When the catalyst concentration within the system exceeds the oxidant, there is scavenging reaction from H_2_O_2_ and H_2_O. The hydroxyl radical, which is very active, is the main radical and it attacks all organic substances in the wastewater. The other radicals (H_2_O_2_, HO_2_^∙^, and O_2_^∙^) are unable to degrade the contaminant.

## 3. Experimental Section

### 3.1. Materials

Chemicals used were of analytical grades and used without further purification. They were hydrogen peroxide (H_2_O_2_, 30 wt%), sulfuric acid (H_2_SO_4_ 97%), sodium hydroxide (NaOH, 50 w/w%), and sodium borohydride (NaBH_4_) from Merck chemical company; and ferrous sulfate (FeSO_4_·7H_2_O) was used from Fisher Scientific Sdn Bhd, Malaysia. FeSO_4_·7H_2_O and NaBH were used for the nZVI preparation and the Fe^0^ obtained was consumed immediately. NaBH_4_ was used to synthesise the catalyst at room temperature (23°C) under Ar protection according to the procedures described by Cheng et al. [28] and Xu and Wang [29]. Briefly, the protocol involved adding 100 mL of aqueous solution of NaBH_4_ (20 mM) to 100 mL of FeSO_4_·7H_2_O (4 mM). The addition was done dropwise with constant violent stirring in a three-necked flask. A stirring time of 90 minutes was allowed. Then, the nZVI deposited was washed thrice with deionised water and vacuum dried.

### 3.2. Fenton Experiment

The mineralisation and degradation were conducted at temperature of 20°C and atmospheric pressure in a 5 L batch reactor. The wastewater's initial pH was adjusted to 3 using NaOH (2 M) or H_2_SO_4_ (2 M). A fixed concentration of Fe^2+^ (Fenton oxidation) or Fe^3+^ (Fenton-like oxidation) was transferred to the reactor containing 4 L of the PRE. To initiate the reaction, H_2_O_2_ was introduced under constant stirring at 200 rpm (to homogenise the mixture). Then, 12.5 mL of the sample was periodically withdrawn at predetermined time interval to follow the extent of mineralisation and degradation with time. Thereafter, the reaction was terminated by spiking the sample with NaOH (2M), which adjusted the pH to 8.5 ± 0.2. This consequently resulted in precipitating iron as Fe(OH)_3_ which would then be filtered using a 0.45 *μ*m filter and subsequently analysed for the COD and TOC. The batch reactions were duplicated and the results obtained suggested reproducibility within an error range of 3%.

### 3.3. Analytical Methods

The chemical oxygen demand (COD) and the total organic carbon (TOC) were determined in the liquid phase of the sampled aliquots by the closed reflux method and oxidative combustion, respectively. For the COD, Hach method number 8000 was adopted where samples were added to Hach vials containing potassium dichromate solution in an acid medium and digested in a HACH DR/200 reactor for 120 min. This action reduces the dichromate ions to chromic ions and subsequently the COD is read from absorbance measurements in a HACH DR/890 colorimeter. The interferences of H_2_O_2_ with COD measurements were eliminated by destroying residual H_2_O_2_ in the treated solution through catalase addition after the pH adjustment.

TOC was measured using a Shimadzu TOC-V CSH analyzer with an infrared detector. Biochemical oxygen demand (BOD) was measured after 5 days of incubation of a microorganism culture according to standard methods.

## 4. Results and Discussion

### 4.1. Mineralization Profile

The extent of mineralisation (TOC reduction) is considered the most suitable to model the kinetic reaction in a myriad wastewater. TOC indicates the extent of conversion of carbon and heteroatoms components in organic compounds to inorganic species. The procedure is much more accurate than COD as it measures the amount of the carbon converted directly. In addition, TOC measurement is not affected by (i) oxidation state of the organic matter; (ii) organically bound elements (nitrogen, hydrogen, and inorganics); and (iii) presence of organics difficult to be oxidized completely [30]. On the other hand, oxygen demands determined by COD tests do not adequately reflect the actual oxygen requirements for oxidation in wastewater with myriad contaminants due to (i) the assumption that all the organic materials can be oxidized by a strong oxidizing agent under acidic conditions by COD procedure—the assertion is not always valid as COD tests have limitation of incomplete oxidation of some aromatic compounds, and (ii) contribution to higher COD values due to easily oxidisable compounds [31]. Typically, PRE contains reduced substances such as ferrous iron, sulphides, and sulphites which are known to be easily oxidised.

For mineralization conditions, the ratio between hydrogen peroxide and organic matter is very significant as the extent of oxidation depends on this parameter, while the oxidation rate is determined by the initial iron concentration [27]. The ratio yielding the lowest concentration of oxidant was chosen in this study to allow for consumption of the reagent and to negate the need for quenching the reagent, as much concentration of H_2_O_2_ might adversely affect a subsequent biological process. Thus, the reagent is gradually reduced after a batch of chemical dosing along with reaction time and most of the reagents would be consumed eventually [12]. In addition, the most effective dosing approach—single dosing, was adopted in the study. Excessive oxidant was used in this method and much of it was left available to be attacked by hydroxyl radicals [12].

As PRE is known to contain varied benzoic groups, the appreciable mineralization observed with nZVI can be attributed to effective mineralization of nitrobenzene (NB) group of which oxidation generates a significant amount of 1,3-dinitrobenzene (1,3-DNB) [32].

This byproduct of NB nitration with nitro radicals (^∙^NO_2_) increases the biodegradability of wastewater. This enhanced treatment is ascribed to two electron-deficient–NO_2_ groups and subsequent mineralization of the generated refractory intermediates of NB hydroxylation [32].

### 4.2. Mineralisation Kinetics

#### 4.2.1. First-Order Kinetic Model (FKM)

The simplest model FKM (5) was examined. All the contaminants in PRE are assumed to be mineralized in only one step, where the OH addition results in the final products (CO_2_ + H_2_O):

(4) $$\begin{array}{c} \text{PRE}\overset{k_{3}^{\prime }}{⟶}{\text{CO}}_{2}+{\text{H}}_{2}\text{O}, \end{array}$$

where *k*_3_′ is the apparent kinetic constant:

(5) $$\begin{array}{c} -r_{{\text{TOC}}_{\text{PRE}}}=-\frac{dC_{{\text{TOC}}_{\text{PRE}}}}{dt}=k_{1}^{\prime }C_{{\text{TOC}}_{\text{PRE}}}. \end{array}$$

**Figure 1:**
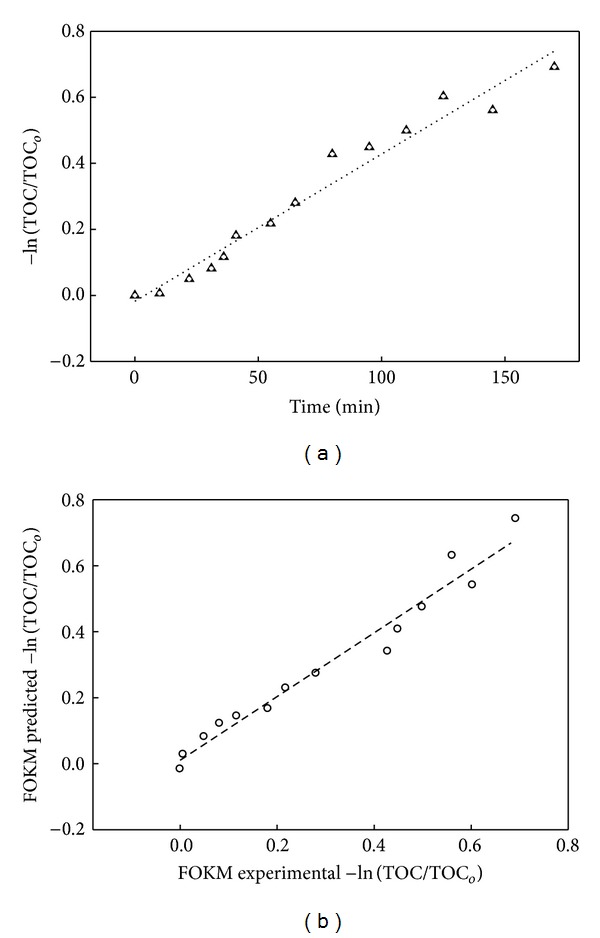
Plots for FKM (a) kinetic and (b) predicted versus experimental.

Based on the plot of −ln⁡⁡(TOC/TOC_*o*_) against time (Figure 1(a)), the apparent first-order kinetic rate constant (*k*_1_′) and the corresponding correlation coefficients (*R* ^2^) of 0.9645 between the experimental and predicted data (Table 1), it is observed that the model could well be used to predict the mineralization process. Thus, the general assumption that the model will not fit *ab initio* due to the limitation imposed by the composition of the wastewater appears irrelevant in our case (for reasons discussed in Section 4.2.2).

Although the *R* ^2^ and the good agreement between the predicted versus experimental plot (Figure 1(b)) suggest explaining the process by the FKM, the limitation imposed calls for further exploring other robust models in addressing kinetic mineralisation of such a complex system, with a vast diversity of possible reaction pathways [13]. Furthermore, as the mineralisation of PRE is attained in multiple stages involving an initial oxidation step leading to transitional conversion of the contaminants to intermediates and followed by subsequent mineralization of the intermediates to the products [9], a simple one-step model may not be sufficient. There is need to explore other models.

#### 4.2.2. Generalized Lumped Kinetic Model (GLKM)

Alumped kinetic model that allows insight into the contribution of PRE → intermediates step, which FKM does not account for, is the simple Generalized Lumped Kinetic Model (GLKM). The simplicity of the model relates to its assumption of negligible influence of PRE to CO_2_ + H_2_O step. To appreciate the need for investigating the intermediates contribution, we briefly looked at this step. It involves addition of the produced ^∙^OH to the aromatic, heterocyclic rings, and unsaturated bonds of alkenes or alkynes [18], thereby enhancing the degradability of aliphatic compounds. However, byproducts are generated along time through partial mineralisation of the other aromatic substances (Figure 1). These intermediates are the results of collapse of aromatic ring during hydroxylation (mineralisation), which yields low molecular-weight carboxylic acids [9, 11, 33].

**Figure 2:**
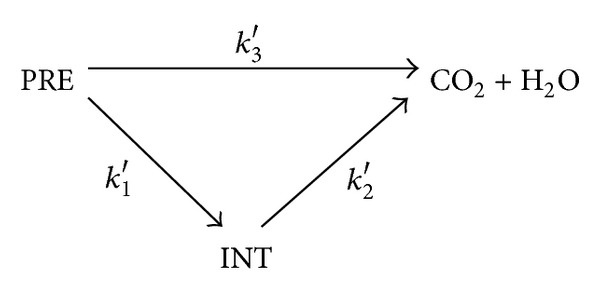
Schematic representation of PRE mineralization, intermediates, and final product.

All of the intermediates that could possibly be produced are lumped into INT, the parent organic substances as PRE, while the final mineralization products are CO_2_ + H_2_O [9, 13]. The generalized degradation schemes and kinetic rate constant are shown in Figure 2, with *k*_1_′, *k*_2_′, and *k*_3_′ as the apparent first-order kinetic rate constants for the initial oxidation step, the final oxidation step, and the direct conversion to endproducts step, respectively.

Equations (6) and (7) describe the degradation of PRE and intermediates, respectively:

(6) $$\begin{array}{c} -r_{{\text{TOC}}_{\text{PRE}}}=-\frac{dC_{{\text{TOC}}_{\text{PRE}}}}{dt}=\left(k_{1}^{\prime }+k_{3}^{\prime }\right)C_{{\text{TOC}}_{\text{PRE}}}, \end{array}$$

(7) $$\begin{array}{c} -r_{{\text{TOC}}_{\text{INT}}}=-\frac{dC_{{\text{TOC}}_{\text{INT}}}}{dt}=k_{1}^{\prime }C_{{\text{TOC}}_{\text{INT}}}-k_{1}^{\prime }C_{{\text{TOC}}_{\text{PRE}}}. \end{array}$$

As the mineralization occurs through the transitional conversion to intermediates followed by subsequent mineralization of the intermediates to the products, *k*_3_′ is then supposedly to be much smaller than *k*_1_′ or *k*_2_′ and thus neglected [9, 34]. Hence, by rearranging (6) and (7), the GLKM becomes

(8) $$\begin{array}{c} \frac{\text{TOC}}{\text{TO}{\text{C}}_{o}}=\frac{k_{1}^{\prime }}{k_{1}^{\prime }+k_{3}^{\prime }+k_{2}^{\prime }}e^{-k_{2}^{\prime }t}-\frac{k_{2}^{\prime }}{k_{1}^{\prime }+k_{3}^{\prime }+k_{2}^{\prime }}e^{-(k_{2}^{\prime }+k_{2}^{\prime })t}. \end{array}$$

The logarithmic form of (9) becomes the following:

(9) $$\begin{array}{c} \ln\frac{\text{TOC}}{\text{TO}{\text{C}}_{o}}=\ln\frac{k_{1}^{\prime }}{k_{2}^{\prime }}-\ln\left(k_{1}^{\prime }+k_{2}^{\prime }\right)t. \end{array}$$

From the plot ln TOC/TOC_*o*_ versus time (Figure 1), the reaction rate constants, *k*_1_′ and *k*_2_′, were found to be 98.36 × 10^−2^ min^−1^ and 97.91 × 10^−2^ min^−1^, respectively. The computed rate constant values were marginally different suggesting that the PRE → intermediates reaction proceeded at the same rate as the intermediates → CO_2_ + H_2_O reaction. This result implies that the hydroxylation of the parent organic contaminants to intermediates cannot be negated.

#### 4.2.3. Generalized Kinetic Model (GKM)

Still, the GLKM imposes a limitation to the full prediction of the stages, as seen in the case of this work. The contribution from KRC in FKM is significant and thus the GKM proposed by Martins et al. [13] was used to further asses the process. It allows the lumping of these chemical pollutants in addition to computing the direct mineralisation of PRE to CO_2_ + H_2_O step. This is a more holistic model as all the steps are accounted for. By rearranging (8) and (9) and taking into account the direct conversion step, we obtain

(10) CTOCCTOCo=CTOCPRE+CTOCINTCTOCPREo+CTOCINTo=CTOCPREoCTOCo(k1′k1′+k3′−k2′e−k2′t+k3′−k2′k1′+k3′−k2′e−(k2′+k1′)t) +CTOCINToCTOCoe−k2′t.

To further simplify the model, recalcitrant intermediates concentration at the initial step is assumed to be nil [13]. Thus, the integrated and normalized form of (11) becomes

(11) $$\begin{array}{c} \frac{\text{TOC}}{\text{TO}{\text{C}}_{o}}=\frac{k_{1}^{\prime }}{k_{1}^{\prime }+k_{2}^{\prime }+k_{3}^{\prime }}e^{-k_{2}^{\prime }t}-\frac{k_{3}^{\prime }-k_{2}^{\prime }}{k_{1}^{\prime }+k_{2}^{\prime }+k_{3}^{\prime }}e^{-(k_{3}^{\prime }+k_{4}^{\prime })t}. \end{array}$$

**Figure 3:**
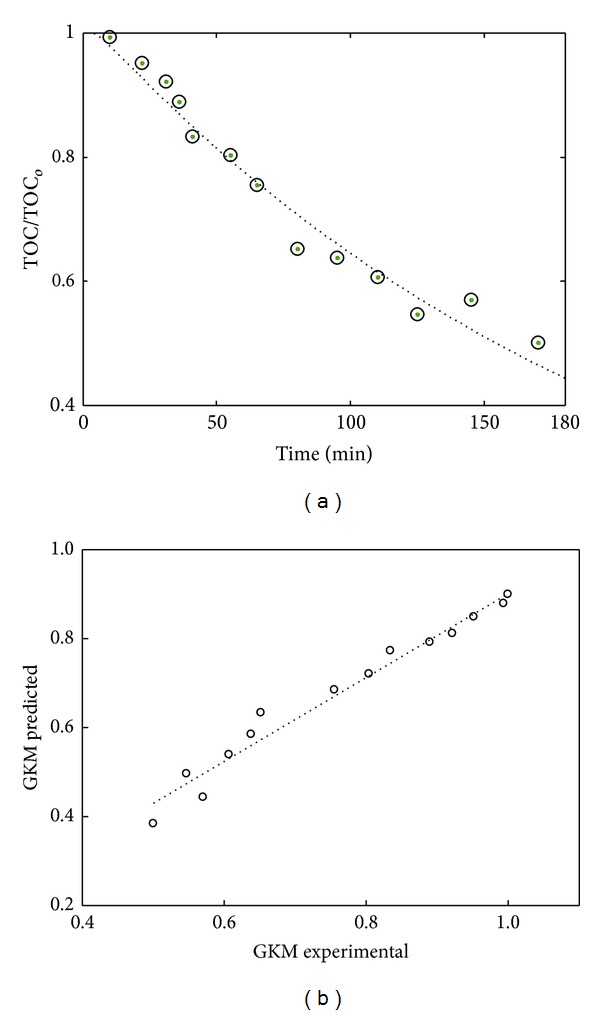
Plots for GKM (a) kinetic and (b) predicted versus experimental.

The plot of (11) and the corresponding predicted and experimental results are depicted in Figure 3.

### 4.3. Goodness-of-Fit Statistics

Four descriptive statistical indicators were used to appraise the prediction performance of proposed models and the produced error in accounting for the kinetic reaction constants and assessing the fit between the experimental data from the models evaluated. The examined indicators were the sum of squares due to error (SSE), *R*-square, adjusted *R*-square, and root mean squared error (RMSE). In the case of GKM, MATLAB was employed (commercial version 7.1) due to complexity of the equation arising from the several constants. The curve fitting toolbox was utilised and, as Levenberg-Marquardt and Gauss-Newton algorithms do not handle bound constraints, trust-region algorithm was implemented.

Based on the results summary (Table 1), it is seen that there are very small deviations in descriptive performance indices of all the models. GKM only marginally varied with FKM and GLKM and demonstrated a slightly superior predictive performance on the estimation of mineralization kinetics. Based on the *R* ^2^ statistic measure, it is evident that the fit successfully accounted for greater proportion of variance as all the models explained *≈*96% of the total variation in the data. Only 3.5% and 2.6% of the total variations were not explained by FKM/GLKM and GKM, respectively. However, as GKM contains more coefficients than the GKLM and FKM, adjusted *R*-square statistic, a generally accepted best indicator of the fit quality in the comparison of models with those that are nested, is used. Moreover, as the *R* ^2^ measured is not an unbiased estimator of the population correlation coefficient, the effect is corrected employing the adjusted correlation coefficient. Again, similar trend was observed as with the *R* ^2^ with GKM being slightly better. Results for SSE show that the total deviation of the response values from the fit-to-the response values measured was acceptable. The lowest values of SSE (0.0104) for GKM against the slightly higher values of SSE (0.0257) for FKM/GLKM indicated that the former model performed better. This indicated that the model had a smaller random error component and that the fit would be more useful for prediction. Lastly, the fit standard error and the standard error of the regression, root mean squared error (RMSE), and the standard deviation of the random component in the data were comparatively better in the case of GKM with RMSE of 0.0323 compared to FKM/GLKM with RMSE of 0.0463.

In summary, the results suggest that all the models adequately describe the kinetics. However, the limitation imposed on FKM and GLKM does not translate to the real-world description of the process. For instance, mineralization was very fast with respect to the other steps, as the kinetic rate constant (*k*_1_′) was 10.12 min^−1^. This clearly indicates that the initial oxidation step leading to transitional conversion of contaminants to intermediates is significantly yet not captured in its entirety in FKM (0 min^−1^) and, in the case of GLKM, a mere 0.98 min^−1^ was computed (representing only 10% of the actual value). This was expected as aromatic degradation which is known to be very fast in contrast to aliphatic degradation [11]. In the same vain, rate constant of the intermediates mineralization to the final products preceded fast too (*k*_2_′). Based on GKM, the rate was *≈*40% higher than that obtained in GLKM and not obtainable using FKM. Finally, the direct conversion of PRE to endproducts step (*k*_3_′) which was not possible to be captured was found to be appreciable. In contract to 4.5 × 10^−3^ min^−1^ estimated by FKM, the actual rate constant was 0.2444 min^−1^, approximately 98% higher than the value obtained by FKM. It is worth noting that mineralization would not have been possible at the rate computed from FKM, especially when steady mineralization was observed within 180 minutes of oxidative treatment period and for fast PRE mineralization.

The use of generalised models becomes necessary in certain reactions. This assertion has been collaborated by several researchers who have elucidated that use of FKM cannot adequately describe the kinetics in systems being continuously fed with H_2_O_2_ [12, 35, 36], for example, monitoring Fenton oxidation in a semicontinuous reactor where the overall amount of H_2_O_2_ is distributed as a continuous feed upon the reaction time [37].

Additionally, reactions not appropriately described by FKM have been addressed by subdividing the reaction period into two or three phases to fit the experimental data using the first-order model separately with different values of kinetic constant (*k*) [38] or use mixed first- and second-order kinetics [39]. This approach may well simulate the experimental data mathematically, but not chemically [12].

This work provides useful information on complex wastewater treatment by heterogeneous nZVI Fenton system, specifically PRE.

## 5. Conclusions

The study presented the kinetic modeling of a nanozerovalent (nZVI) heterogeneous Fenton oxidative mineralization of petroleum refinery effluent (PRE). The oxidation specific variable data was generated at constant pH of 3.0 and fixed ratios of 2 and 20 for H_2_O_2_ : PRE and H_2_O_2_ : Fe^0^, respectively. The data was fitted to three different models, first-order kinetic model (FKM), generalised lumped kinetic model (GLKM), and generalized kinetic model (GKM).

Based on the results obtained and the four descriptive statistical indicators used for appraisal of the prediction performance of proposed models, only small deviations were observed and the data fitted satisfactorily generally. Although GKM demonstrated a slightly superior predictive performance on the estimation of mineralization kinetics in comparison to FKM and GLKM, the corresponding predictive kinetic rate values varied significantly. With the most significant step being the initial oxidation step leading to transitional conversion of contaminants to intermediates, only 10% of the actual value was captured by GLKM and the limitation of FKM does not allow for determining the contribution of this step. The second fastest step was the intermediates mineralization to the final products (*k*_2_′). Again, the rate predicted by GLKM was *≈*40% lower than the value obtained in GLKM and naturally not obtainable using FKM. Considering that GKM is able to predict simultaneously all the steps of PRE hydroxylation, it is deemed to be the most suitable model.

This work provided useful information on complex wastewater treatment kinetics by heterogeneous nZVI Fenton system, specifically PRE.

## Figures and Tables

**Table 1:** FKM, GLKM, and GLKM kinetic rate constants and statistical data.

Parameter	Kinetic models		
	FKM	GLKM	GLKM
Kinetic rate constants			
*k*_1_′ (min^−1^)	na	0.9836	10.1248
*k*_2_′ (min^−1^)	na	0.9791	0.2444
*k*_3_′ (min^−1^)	0.0045	ng	3.7852
Statistical indicators			
*R* ^2^	0.9650	0.9650	0.9736
*R*_adj_^2^	0.9621	0.9621	0.9660
SSE	0.0257	0.0257	0.0104
RMSE	0.0463	0.0463	0.0323

na: not applicable; ng: negligible.
